# Comparative Mapping and Candidate Gene Analysis of *SSIIa* Associated with Grain Amylopectin Content in Barley (*Hordeum vulgare* L.)

**DOI:** 10.3389/fpls.2017.01531

**Published:** 2017-09-05

**Authors:** Xiangyun Fan, Juan Zhu, Wenbin Dong, Yuandong Sun, Chao Lv, Baojian Guo, Rugen Xu

**Affiliations:** Jiangsu Key Laboratory of Crop Genetics and Physiology, Co-Innovation Center for Modern Production Technology of Grain Crops, Key Laboratory of Plant Functional Genomics of the Ministry of Education, Barley Research Institution of Yangzhou University, Yangzhou University Yangzhou, China

**Keywords:** barley, amylopectin, QTL, GWAS, GBS, *SSIIa*

## Abstract

Amylopectin concentration in barley endosperm has important effects on grain quality and end-use. In this study, quantitative trait locus (QTL) analysis together with genome-wide association studies (GWAS) were performed to identify markers linked to grain amylopectin content respectively using a doubled haploid (DH) population of 178 lines and a collection of 185 diverse barley germplasms both genotyped by genotyping-by-sequencing (GBS). A stable QTL on chromosome 7H and 11 associated single nucleotide polymorphisms (SNPs) were detected. In the co-localized region, the *SSIIa* (*SSII-3*) gene was predicted as the candidate gene. Then we isolated and characterized biparental *SSIIa* alleles of the DH population, investigated the expression pattern by quantitative real-time PCR (qRT-PCR), and revealed that a 33-bp deletion in exon 2 is responsible for reducing *SSIIa* transcript, thus resulting in a reduced amylopectin content. A sequence-based molecular marker was developed for the *SSIIa* allele and validated the effectivity, which would provide help for barley breeding.

## Introduction

Barley (*Hordeum vulgare* L.) is the fourth largest cereal crop planted worldwide with the extensive utilization in various fields. Barley grain has been widely used as livestock feed and raw material for malting and brewing, while it is also used as the major food source in some regions around the world and appreciated as a component of a healthy diet (Baik and Ullrich, 2008; Newton et al., 2011; Ullrich, 2011). Starch, of which amylose and amylopectin are the two components, is the main constituent in barley grain like other cereal grains (James et al., 2003; Zeeman et al., 2010; Asare et al., 2011). The proportion of amylose to amylopectin in barley endosperm affects certain properties of the starch which may further influence the grain malting, food and feed quality as well as the product application (James et al., 2003; Jaiswal et al., 2010; Jane et al., 2010). As compared to normal starches, high-amylose starches with the high amylose/amylopectin ratio are usually used for producing resistant starches and the products with high gelling strength in related industries, while amylose-free starches with the low amylose/amylopectin ratio generally show the suitability for frozen foods industrial applications because of the excellent freeze-thaw stability (Bird et al., 2000; Jobling, 2004; Jaiswal et al., 2010; Jane et al., 2010).

ADP-glucose Pyrophosphorylase (AGPase), granule-binding starch synthase (GBSS), soluble starch synthase (SSS), starch branching enzyme (SBE), and debranching enzyme (DBE) are known as the 5 classes of enzymes involving in the starch biosynthesis. Of these, GBSS as the key enzyme participates in amylose synthesis, while SSS, SBE, and DBE are believed to have unique functions and concerted actions in amylopectin synthesis (James et al., 2003; Fan et al., 2016). Each class of these enzymes has several isoforms encoded by the related genes, furthermore, each relevant gene has multiple alleles with different genetic effects. Starch synthase IIa (SSIIa) encoded by *SSIIa* (*SSII-3*) gene is the one of a family enzymes that work progressively in the conversion of ADP-glucose to starch polymers by elongating short amylopectin chains which has the degree of polymerization (DP) ≤ 10 to intermediate chains of amylopectin (DP = 12~24) in the cereal endosperm (Fontaine et al., 1993; Luo et al., 2015). It has been reported in the barley, wheat, rice and maize that the composition and content as well as properties of grain starch can be changed by the altered SSIIa (Yamamori et al., 2000; Umemoto et al., 2002; Morell et al., 2003; Zhang et al., 2004; Konik-Rose et al., 2007).

Understanding the molecular and genetic mechanism of amylopectin is important in barley starch quality improvement. However, nearly all the studies in barley about amylopectin synthesis enzymes/genes were conducted with mutant materials. Up to now, there are no reports on the study about detecting QTL for barley grain amylopectin concentration via linkage mapping analysis. Furthermore, there is only one report on GWAS for grain amylopectin content in barley, which identified 17 associated single nucleotide polymorphisms (SNPs) using a collection of 254 European spring barley varieties (Shu and Rasmussen, 2014).

In this study, we mapped QTLs responsible for amylopectin content in barley by using a bi-parental population together with an association mapping panel both already genotyped with SNPs developed by genotyping-by-sequencing (GBS). Identification of the relevant QTL by the combination of linkage analysis and GWAS has not been reported before. Furthermore, the candidate genes in the mapping region were predicted and analyzed by the annotation according to syntenous and comparative genomics with rice (*Oryza sativa* L.). The *SSII-3* (*SSIIa*) gene was subsequently characterized and markers related to the gene were designed and validated. The results of this study reveal new genetic insights into grain amylopectin content in barley and provide the sequence-based marker for use in marker-assisted selection (MAS) for barley breeding.

## Materials and methods

### Plant materials

This study was performed by using two sets of plant materials. For the linkage mapping analysis, a bi-parental population of 178 DH lines was generated from a cross of barley (*Hordeum vulgare* L.) cultivars TX9425 and Naso Nijo. Both TX9425 and Naso Nijo are two-rowed winter barleys, with TX9425 (Chinese feed barley) having low malting quality but excellent resistance and tolerance and Naso Nijo (Japanese malting barley) having the opposite characteristics (Pang et al., 2004; Li et al., 2008; Xu et al., 2012). For the GWAS study, 185 winter barley (*Hordeum vulgare* L.) varieties were collected as the association mapping panel (Table S1). The panel comprises 97 two-rowed and 88 six-rowed types, and among them 164 accessions originate from China, 9 from Japan, 8 from USA, 2 from Australia, 1 from UK and 1 from Hungary. Most of the accessions are hulled barley (160), the other are naked barley (25). All of the plant materials had been genotyped with SNPs via GBS by USDA-ARS and Agronomy department of K-State. Growth condition including the planting location and year of the two mapping materials as well as the field experimental design and agronomic management were previously described in detail in Fan et al. (2017). After harvesting, the mature grains were air-dried and then about 10 g of grains for each line/variety were milled into flour. All the sample flours were further passed through 100 (0.15 mm) screen and stored in sealed plastic bags in the 4°C fridge before use.

### Measurement of amylopectin content

The amylopectin content of barley grain was measured by the method of Gibson et al. (1997). Firstly, the total starch content of the barley grain was measured by the Megazyme Total Starch Assay kit (K-TSTA, Megazyme, Ireland), then the proportion of amylose in total starch was determined by the Megazyme Amylose/Amylopectin Assay Kit (K-AMYL, Megazyme, Ireland) and the amylopectin content of barley grain was calculated by the difference between total starch and amylose content. For each independent sample, the analyses were conducted in triplicate.

### Statistical analysis and QTL and GWAS mapping

Analysis of variance (ANOVA) of amylopectin content was conducted by Matlab v7.0, and descriptive statistical analysis, frequency distribution analysis and *T*-test analysis were implemented using SPSS 16.0 Statistic software.

QTL and GWAS analyses were carried out as detailedly described in a recent study of Fan et al. (2017). For the DH population, JoinMap v4.0 (Van Ooijen, 2006) together with QTL IciMapping v4.0 (Wang, 2009; http://www.isbreeding.net/software/) were employed to construct the genetic linkage map and perform the QTL detection and analysis. The mean amylopectin content values of two locations and the values of each location were all used in QTL identification. The detected QTL was only regarded as the valid locus when it was identified in at lowest two different environments. For the association mapping panel, the population structure (Q) and the kinship matrix (K) were respectively analyzed by Structure 2.3.4 (Pritchard et al., 2000) and TASSEL 5.0.9 (Bradbury et al., 2007) using filtered 3,826 SNP markers (missing date <20%, minor allele frequency (MAF) >5%, heterozygosity rate <5% and with positional information in barley cultivar Morex reference genome). Best linear unbiased predictors (BLUPs) was chosen to calculate out the value which was used in the subsequent analyses from the phenotypic amylopectin content value of 2 years. Association analysis was conducted with TASSEL 5.0.9 (Bradbury et al., 2007) followed by the MLM with Q and K model which had been evaluated the suitability for this study. The critical *P*-value for evaluating the significance of marker-trait-associations (MTA), which was too stringent evaluated by the false discovery rate (FDR), was declared at 0.001 via a liberal method by Chan et al. (2010).

### Gene prediction and DNA sequence analysis

In this study, IPK Barley BLAST Server (http://webblast.ipk-gatersleben.de/barley/) together with Plant Genome and Systems Biology (PGSB) (http://pgsb.helmholtz-muenchen.de/plant/barley/gz/download/index.jsp) and The Rice Annotation Project Database (RAP-DB) (http://rapdb.dna.affrc.go.jp/) were utilized to search for location information of the identified marker, download the genes within the QTL region, and predicted candidate genes by the annotation based on syntenic to rice. A CTAB method of Stein et al. (2001) was used to prepare the fresh leaf genomic DNA samples in this study. Multi-primers designed by Primer Premier 5.0 were used to isolated and characterized the predicted gene in bi-parental genome (TX9425 and Naso Nijo) of the DH population (For details see Fan et al., 2017).

### Marker development and validation analysis

The sequence-based molecular marker was designed by Primer Premier 5.0 based on the result of sequence analysis. Subsequent allele-specific genotype detection by using the developed marker was carried out in the leaf DNA samples of 185 barley varieties consisted in the association mapping panel as well as in the cDNA samples obtained from the bi-parental developing seeds of the DH population.

### Quantitative real-time Pcr analysis

Total RNA was isolated from the diverse days after flowering (DAF) (5, 10, 15, 20, 25, and 30 DAF) seeds and used to generate the cDNA. The details of the quantitative real-time PCR (qRT-PCR) analysis had been described in a recent study of Fan et al. (2017).

## Results

### Phenotypic analysis

**Figure 1:**
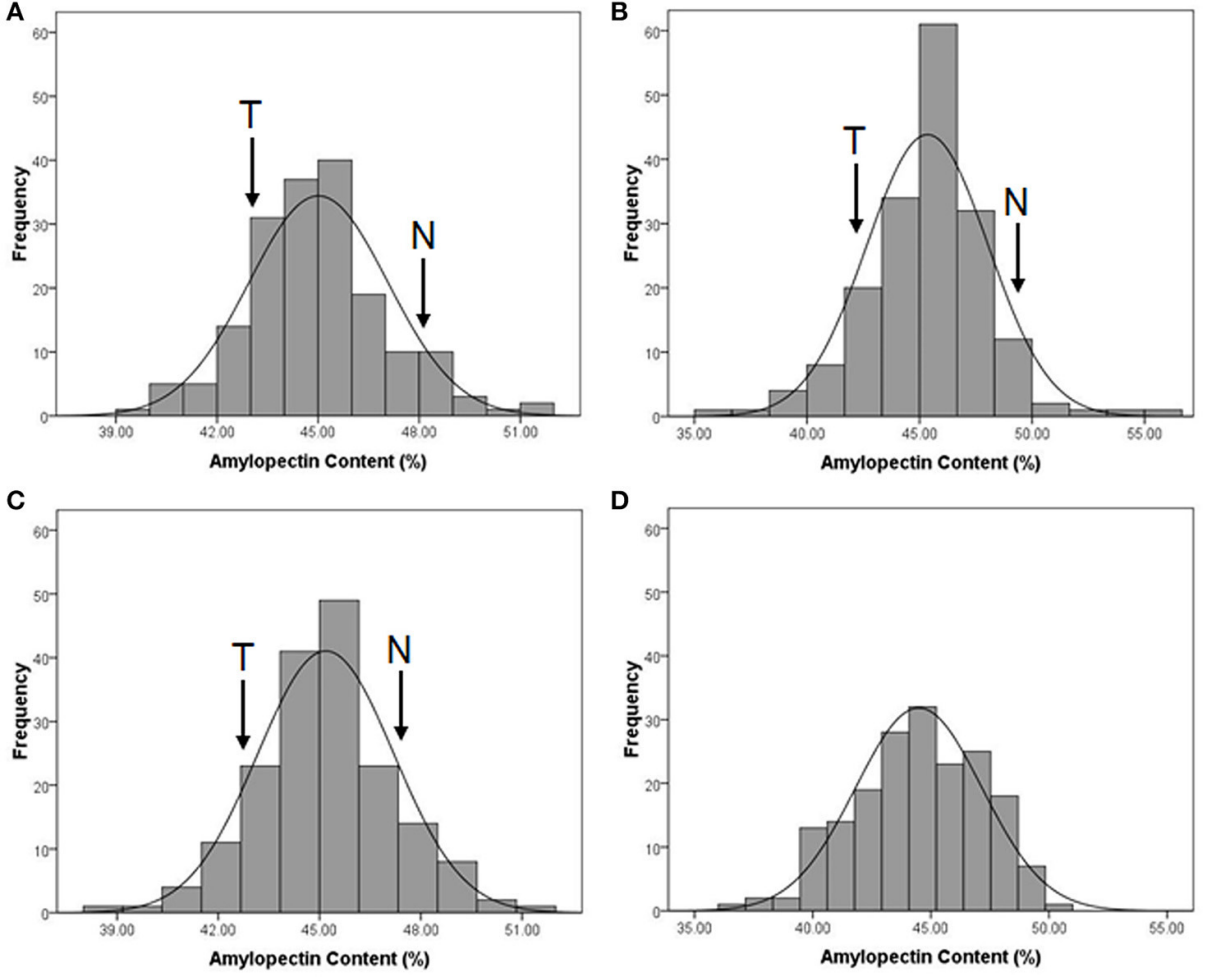
The performance of grain amylopectin content in two populations. **(A)** The amylopectin content performance of TX9425/Naso Nijo DH population in 2013-2014, Yangzhou; **(B)** The amylopectin content performance of TX9425/Naso Nijo DH population in 2014-2015, Yancheng; **(C)** The amylopectin content performance of TX9425/Naso Nijo DH population getting from means of two environments; **(D)** The amylopectin content performance of the population of 185 barley varieties getting from BLUPs of 2 years.

The distribution of amylopectin content in the DH population with the range from 39.77 to 51.51% and 35.32 to 55.42% respectively in two environments were both continuous and relatively normal (Figures 1A,B; Table S2). In addition, the distribution of amylopectin content in the association mapping panel with the range from 36.49 to 50.19% showed the similar condition which was continuous and relatively normal (Figure 1D). In the bi-parental population, Naso Nijo had the higher grain amylopectin content compared with TX9425 in each different environment (Figures 1A–C; Table S2). Furthermore, the differences among genotypes and effects of environments were significant in both the bi-parental population (Table 1) and the association mapping panel (Table 2).

**Table 1:** ANOVA of grain amylopectin content in DH population.

	***df***	**Amylopectin content**	
		***MS***	***F***
Genotype(G)	177	21.77	11.51^**^
Environment(E)	1	16.73	8.84^**^
G × E	177	13.00	6.87^**^
Error	710	1.89	

** *Indicate significant at 1% level (P < 0.01)*.

**Table 2:** ANOVA of grain amylopectin content in the collection of 185 barley varieties.

	***df***	**Amylopectin content**	
		***MS***	***F***
Genotype(G)	184	61.65	53.76^**^
Year(Y)	1	241.30	210.42^**^
G × Y	184	10.21	8.90^**^
Error	740	1.83	

** *Indicate significant at 1% level (P < 0.01)*.

### QTL mapping by linkage analysis

The genetic linkage map of the DH population was constructed with a total of 1,551 SNP markers (Figure S1). The markers were uniformly distributed along the chromosomes and the whole map spanned 957.09 cM on 7 linkage groups with an average distance of 0.61 cM between two neighboring markers. Chromosome 3H contained the largest number of markers (up to 378), while chromosome 6H had only 105 markers.

**Figure 2:**
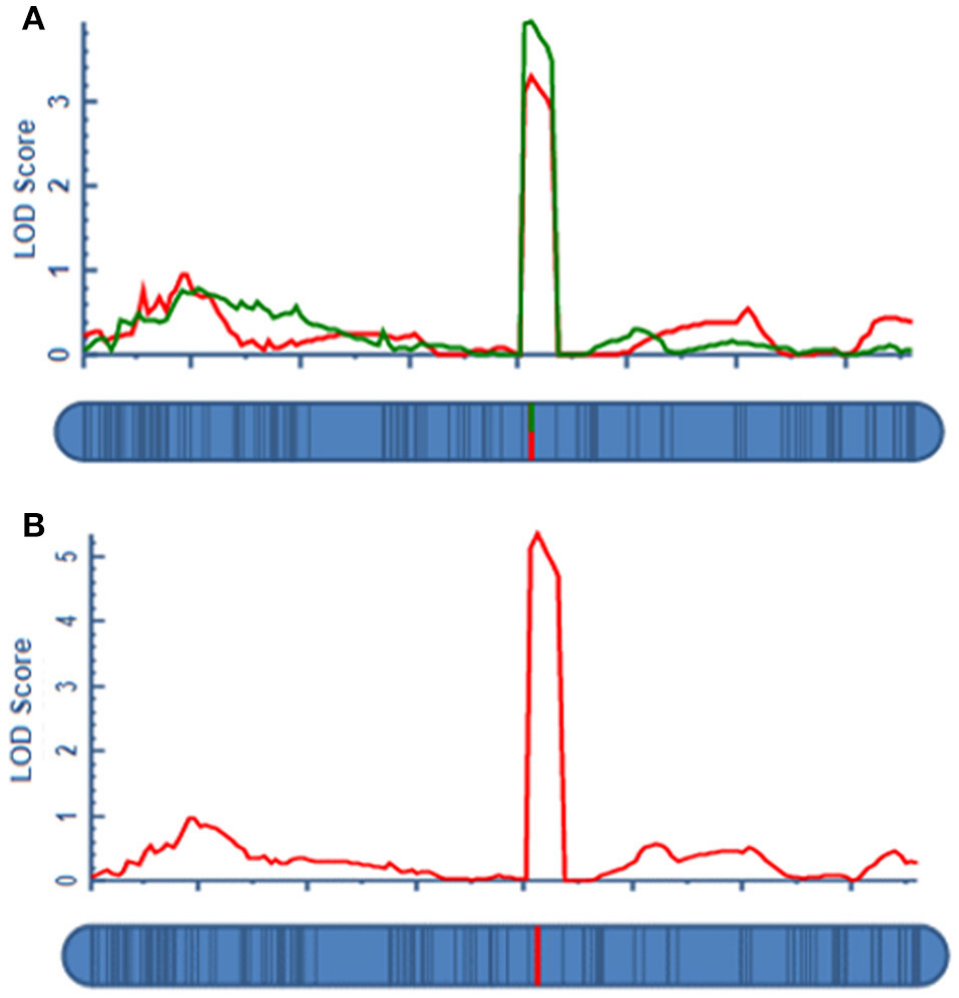
The QTL for grain amylopectin content detected in the DH population. **(A)** The stable QTL on 7H in two environments for amylopectin content; **(B)** The QTL on 7H identified by the means of two environments.

A total of four QTLs were detected in two environments, namely, *qAPC-3-1, qAPC-4-1, qAPC-5-1*, and *qAPC-7-1* (Table 3) from the bi-parental population evaluated with a 2.0 LOD threshold. However, only one QTL, *qAPC-7-1* with the closest marker of SNP2508 (81.79 cM) (67.63 cM in the barley cv. Morex reference genome) was detected in both two environments (Table 3; Figure 2A), explaining the genetic variation of 8.20 and 10.33%, respectively. The LOD value as well as the phenotypic variation could respectively increase to 5.32 and 12.98% analyzed by using the mean amylopectin content values of two environments (Table 3; Figure 2B).

**Table 3:** QTLs analysis for grain amylopectin content detected in the DH population.

**Trait**	**Environment**	**QTL**	**Chr**.	**Position(cM)^a^**	**Linked Marker**	**LOD**	**R^2^(%)**	**ADD(%)**
Amylopectin Content	Yangzhou (2013-2014)	*qAC-3-1*	3H	155.9(154.1)	SNP1920	2.18	5.07	0.47
		*qAC-7-1*	7H	81.8(67.6)	SNP2508	3.28	8.20	−0.64
	Yanchen (2014-2015)	*qAC-4-1*	4H	41.5(54.2)	SNP0788	2.45	6.32	0.63
		*qAC-5-1*	5H	171.4(167.9)	SNP2137	2.64	7.36	0.67
		*qAC-7-1*	7H	81.8(67.6)	SNP2508	3.93	10.33	−0.85
	Mean	*qAC-3-1*	3H	155.9(154.1)	SNP1920	2.11	4.66	0.44
		*qAC-7-1*	7H	81.8(67.6)	SNP2508	5.32	12.98	−0.78

a *The position (within parenthesis) in reference genome of cv.Morex*.

### QTL mapping through GWAS

**Figure 3:**
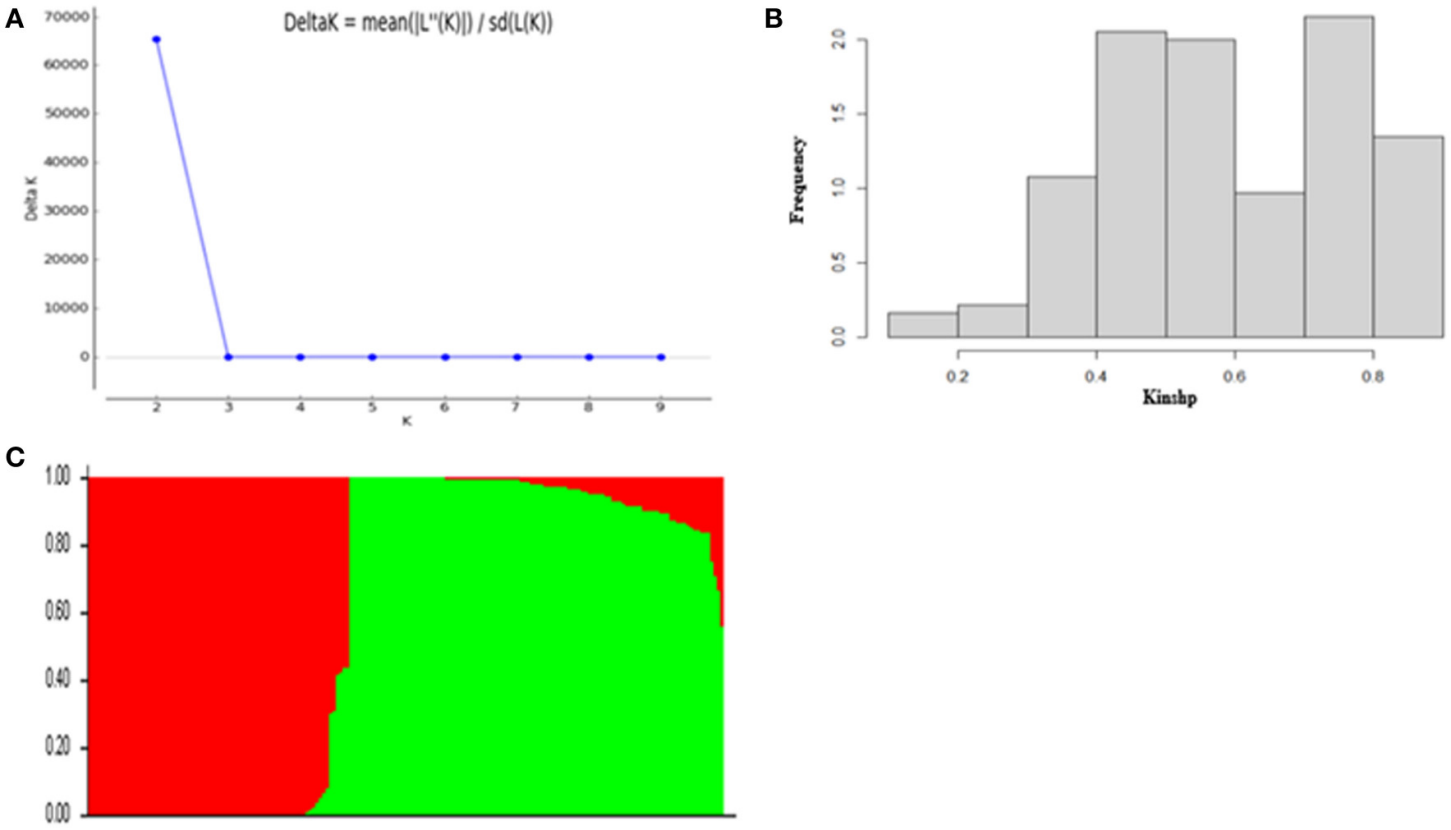
Population structure and kinship analysis of the population of 185 barley varieties based on 3,826 SNPs. **(A)** Δk as a function of the number of subpopulations (k); **(B)** Distribution of relative kinship coefficient across the 185 barley varieties; **(C)** Genetic structure based on Bayesian model.

**Figure 4:**
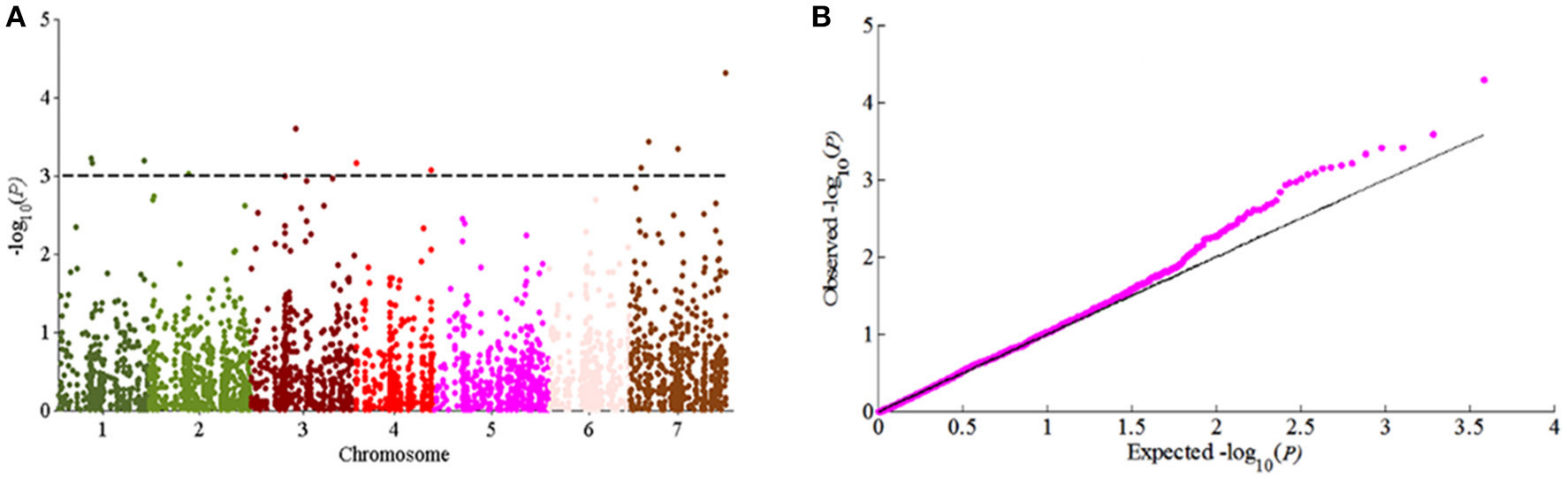
GWAS of grain amylopectin content. **(A)** Manhattan plots of the MLM model for amylopectin content; **(B)** Quantile-quantile plot of the MLM model for amylopectin content.

According to the results of population structure and familial relationship analyses, two subgroups and about 0.5 frequency of kinship were evaluated (Figure 3). Moreover, as shown in the quantile-quantile (Q-Q) plot (Figure 4B), the MLM model incorporating Q and K was suitable for this study as the effect of the population structure on amylopectin content was reduced by using this model. Eleven SNP markers responsible for grain amylopectin content were detected by the threshold of *P* < 0.001 (Table 4; Figure 4A). These significant SNPs were located on 1, 2, 3, 4, and 7H and explained 7.4–12.4% of the phenotypic variation. Of the identified significant SNPs, SNP3120, which was located on chromosome 7H at 70.68 cM (in the barley cv. Morex reference genome), was in a similar position to that identified from the linkage mapping population. Furthermore, SNP3210 was also detected in the analysis for single year separately (Table S3).

**Table 4:** Associated SNPs for grain amylopectin content in the collection of 185 barley varieties.

**Traits**	**Marker**	**Chr**.	**Position(cM)^a^**	***P*-value**	**R^2^(%)**	**Annotation**
Amylopectin Content	SNP1472	1	47.82	6.07E-04	8.87	NA
	SNP0336	1	49.58	6.85E-04	9.68	NA
	SNP0057	1	126.06	6.48E-04	9.16	NA
	SNP2489	2	59.35	9.48E-04	8.45	MLOC_6548.1, Zinc finger protein
	SNP0600	3	68.20	2.52E-04	11.47	MLOC_26433.1, maternal effect embryo arrest 22
	SNP2706	4	19.63	7.05E-04	8.83	MLOC_1888.2, unknown protein
	SNP4404	4	111.96	8.55E-04	8.37	AK353728, UDP-N-acetylglucosamine–peptide N-acetylglucosaminyltransferase
	SNP4298	7	15.93	8.05E-04	7.43	NA
	SNP3654	7	26.71	3.76E-04	11.03	MLOC_62313.1, Protein-O-fucosyltransferase 1
	SNP3120	7	70.68	4.52E-04	9.26	MLOC_62313.1, Endo-1,4-beta-glucanase
	SNP3630	7	140.86	4.96E-05	12.44	NA

a *The position in reference genome of cv.Morex*.

### Candidate genes for amylopectin contents

**Figure 5:**
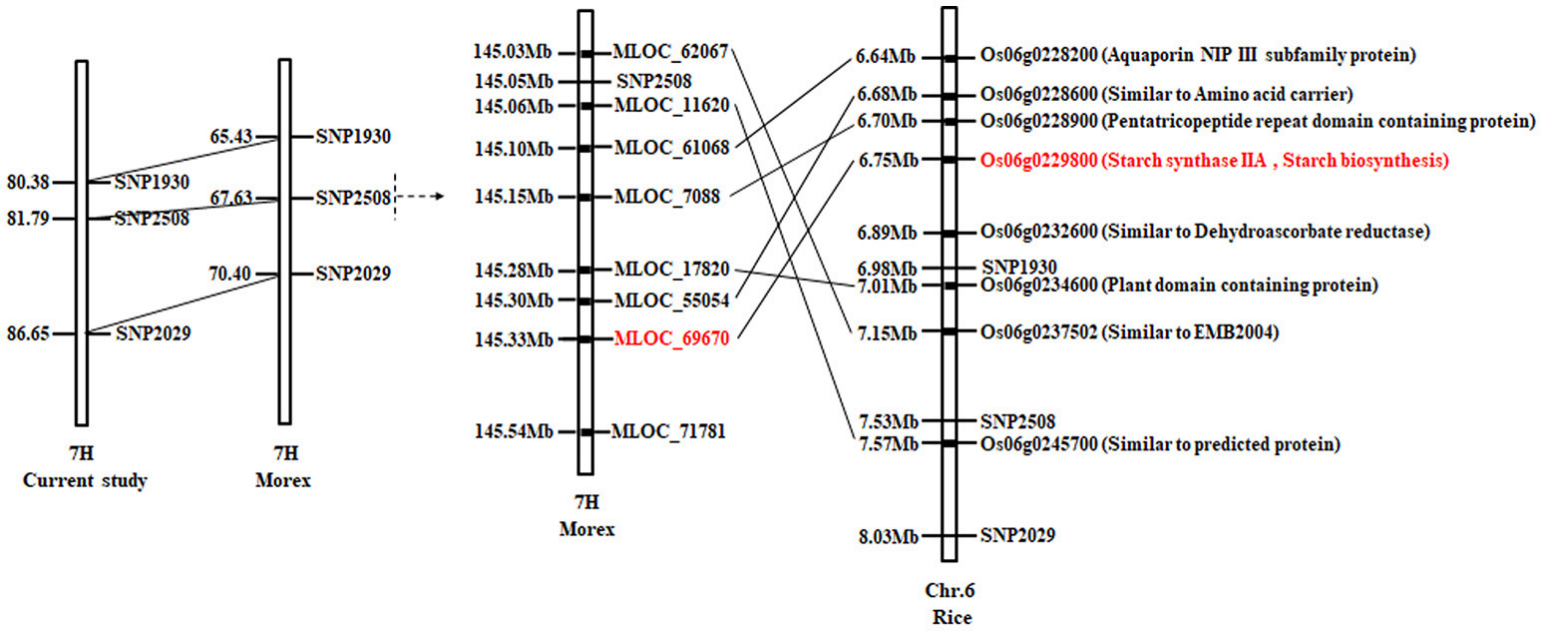
Gene prediction according to syntenous and comparative genomics with rice.

As shown in Figure 5, the QTL mapping interval of *qAPC-7-1* in our study is 80.38–86.65 cM on 7H, while it is 65.43–70.40cM on 7H compared to that in barley cv. Morex reference genome. The region around linked marker SNP2508 of *qAPC-7-1* shows a synteny on rice Chr. 6, and candidate genes around SNP2508 are annotated according to orthologous of Oryza sativa Japonica (Figure 5). Among annotated genes, the gene (*Os06g0229800*) encoding starch synthase IIa is *SS*II*a* (*SS*II*-3*) gene which is known for playing the important role in amylopectin synthesis in grain endosperms (James et al., 2003). Therefore, the *SS*II*a* gene is predicted as the candidate gene of *qAPC-7-1*.

**Figure 6:**
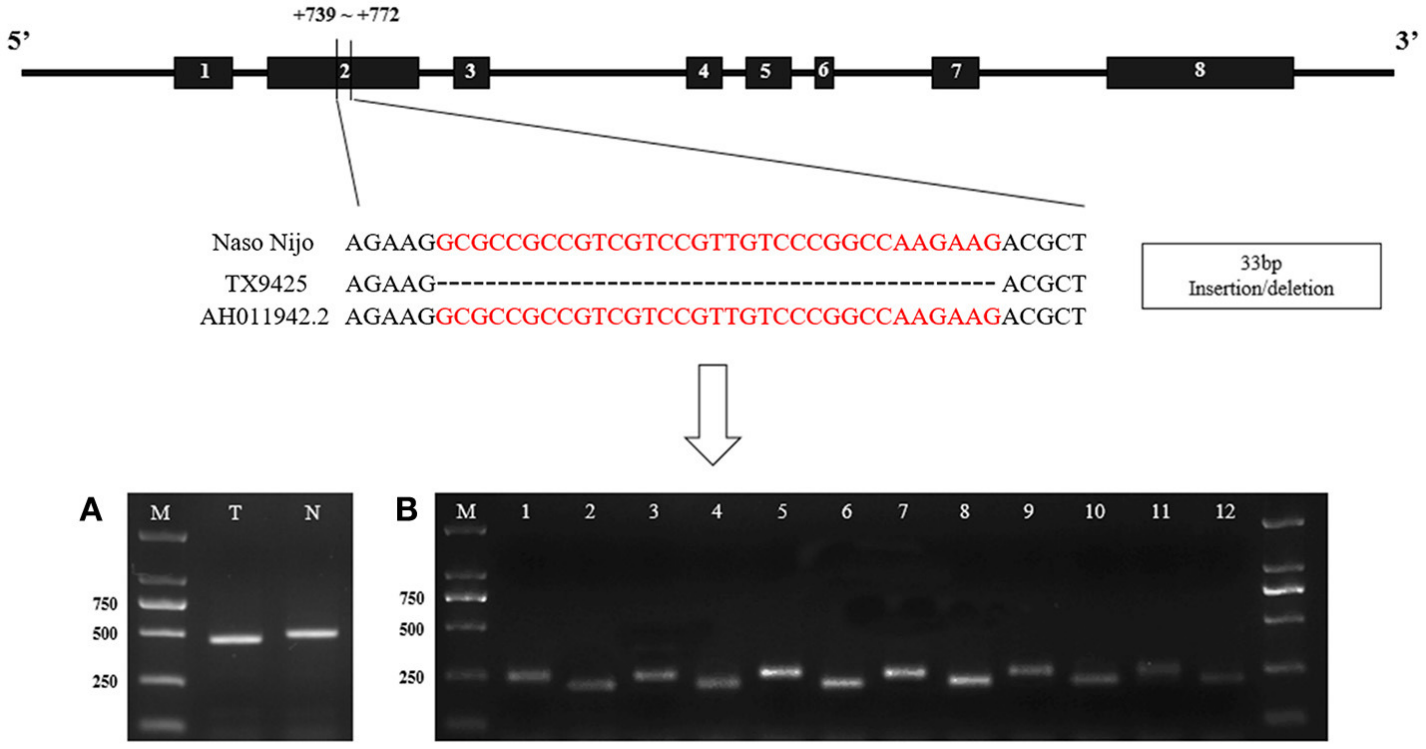
The structure of barley *SSIIa* (*SSII-3*) gene and the nucleotide difference between two parents. **(A)**. Identification of the 33-bp nucleotide difference between two parents in DNA. N, Naso Nijo; T, TX9425; M, 2,000 molecular marker; **(B)** Identification between two parents in cDNA of diverse DAF seed. 1, 3, 5, 7, 9, 11, cDNA of Naso Nijo in 5-d intervals DAF seed; 2, 4, 6, 8, 10, 12, cDNA of TX9425 in 5-d intervals DAF seed. AH011942.2, genbank accession of the gene sequence in NCBI.

The *SS*II*a* alleles sequences without intron 7 of two bi-parental varieties (TX9425 and Naso Nijo) of the linkage mapping population was isolated and characterized by using 5 pairs of primers (SSIIa-1~ SSIIa-5) (Table S4). And then a 33-bp insertion/deletion in exon 2 was identified according to the result of sequencing and alignment (Figure 6).

**Figure 7:**
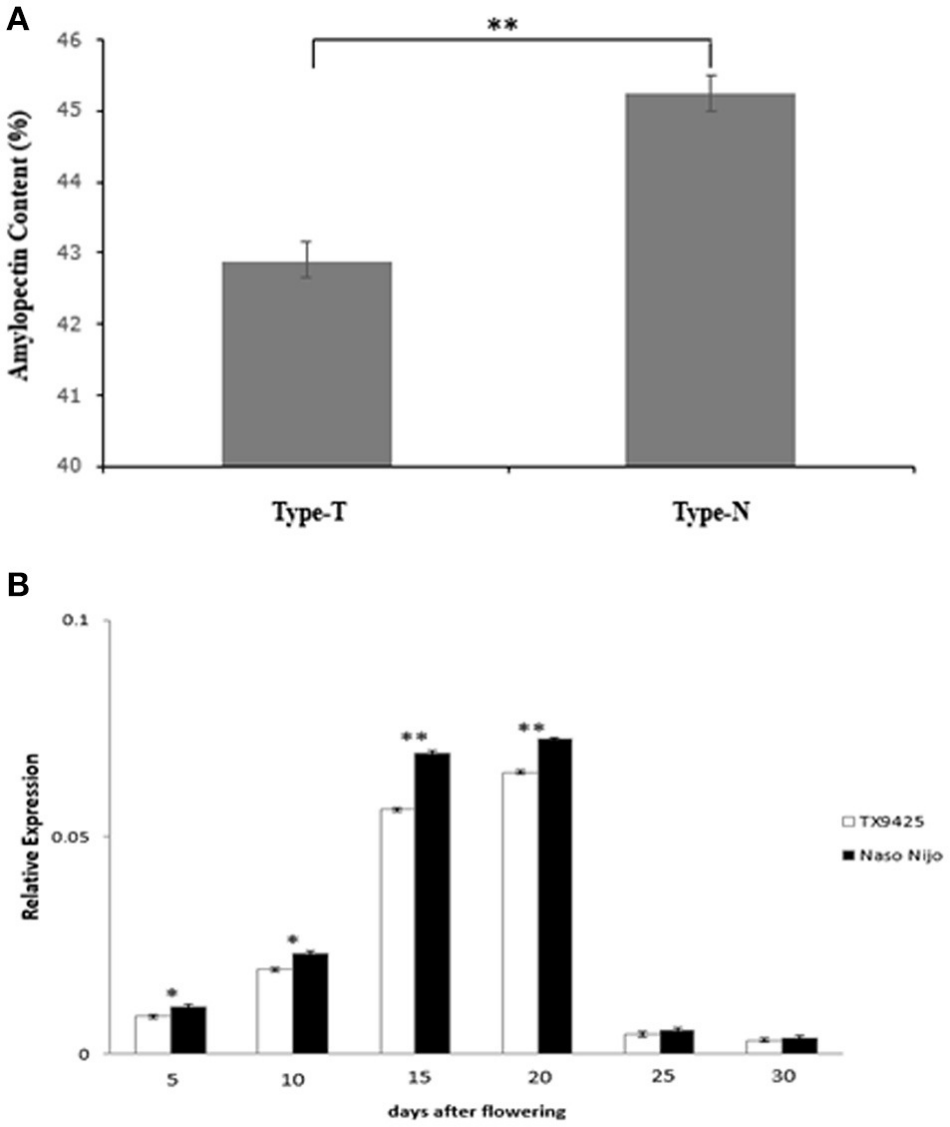
Related validation analysis of *SSIIa* (*SSII-3*) gene. **(A)** Difference in amylopectin content between two types amplified by primer SSIIa-DNA. Type-T, TX9425; Type-N, Naso Nijo; **(B)** Expression of *SSIIa* alleles of two parents in diverse DAF seed. ^*^Indicate significant at 5% level (P < 0.05); ^**^Indicate significant at 1% level (P < 0.01).

Detected by the allele-specific primers of SSIIa-DNA and SSIIa-RNA, respectively, the 33-bp nucleotide polymorphic difference could be found both in DNA and cDNA between the two bi-parental varieties (TX9425 and Naso Nijo) (Figure 6; Table S4). Removing 3 varieties with no amplified band, 185 barley varieties of the association mapping panel were genotyped into two types (58 type-TX9425 and 124 type-Naso Nijo) performed using SSIIa-DNA. Furthermore, as shown in Figure 7A, the difference between the two types was significant in the grain amylopectin content.

A gene-specific primer SSIIa-RT (Table S4) was designed and used to estimate the *SS*II*a* expression pattern in the diverse developing seeds by qRT-PCR analysis. As shown in Figure 7B, the expression levels of the two *SS*II*a* alleles showed the same tendency of low at early seed development, increasing from about 13 DAF, highest at about 15–20 DAF and then decreasing rapidly. Moreover, the expression level of the *SS*II*a* allele in Naso Nijo was significant higher than that in TX9425 at the first four DAF stages.

## Discussion

The main constituent of barley grain is starch which comprises the two components of amylose and amylopectin. Variations in amylose/amylopectin content and amylopectin structure can affect starch properties which may further influence grain qualities for different utilizations (James et al., 2003; Jaiswal et al., 2010; Jane et al., 2010). As to measuring the amylopectin content, the colorimetric method is used relatively easily and inexpensively, however, the problem experienced in the use of this method has been detailed by Gibson et al. (1997) and the method extensively used in the amylopectin content measurement is through the difference calculation between total starch content and amylose content (Shu and Rasmussen, 2014). In this study, we use the Megazyme total starch assay kit together with the Megazyme amylose/amylopectin assay kit (a modification of a Con A method) to measure the amylopectin content phenotype.

Almost all the quality-related phenotypes in the grain crop are the quantitative trait which was generally studied via QTL or GWAS mapping to identify specific gene/locus. The complementarity of classical linkage analyses and genome-wide association study has been well demonstrated by Brachi et al. (2010). Furthermore, the developing sequencing technologies has greatly increased the SNPs discovery in many species (Davey et al., 2011). GBS is a relatively low cost, simple and efficient approach for discovering and genotyping the sample with genome-wide SNP markers (Elshire et al., 2011; Poland and Rife, 2012). High-density genetic maps constructed by SNP markers through GBS have great value for applications in crop breeding and genetics research. In the present study, the grain amylopectin content trait of 178 DH lines and 185 barley varieties, which were all genotyped with SNPs developed via GBS, was measured in diverse environments.

As easily influenced by multiple factors, most of QTLs relevant to quantitative traits were detected only under a few environmental conditions or with the small effect. Hence, the QTL identified stably in various environments and genetic backgrounds as well as with the large effect could be effectively used in MAS for breeding. In our study, a total of four QTLs on chromosome 3, 4, 5, and 7H were detected in two locations from the DH population (Table 3). Among them, *qAPC-7-1* was identified in both environments and determined 12.98% of phenotypic variation (Table 3; Figure 2A). In addition, *qAPC-7-1* was co-localized with an associated SNPs (SNP3120) detected by GWAS from the collection of 185 barley varieties. According to Comadran et al. (2012), and considering a LD of 4-6 cM in barley, the associated SNP3210 on chromosome 7H located at 70.68 cM (in the reference genome of cv. Morex) (Table S5), which was co-localized with *qAPC-7-1*, was located just near the starch synthase IIa gene (MLOC_69670, *SS*II*a*), also designated as *SS*II*-3*. 17 SNPs associated with grain amylopectin content were identified by GWAS in the report of Shu and Rasmussen (2014). Among them, one significant SNP region on 7H (5 SNPs from 12.75 to 15.37 cM) was overlapping with SNP4298 (15.93 cM on 7H) detected in our study. The region was very close to the *waxy* locus which was known to be essential in amylose synthesis and metabolism. Although the accumulated knowledge indicated that the *waxy* gene is the key gene mainly responsible for amylose content, it also has influence on amylopectin structure and content (Denyer et al., 1996; Hori et al., 2007).

The starch biosynthesis pathway has been extensively and deeply researched in recent decades. *SS*II*a* (*SS*II*-3*), which is known as an important gene in amylopectin biosynthesis, has the significant effect on starch content and quality (James et al., 2003; Nakamura et al., 2005; Fan et al., 2016). In our study, candidate genes relevant to amylopectin synthesis were predicted in the co-localized mapping region based on the gene annotation. The gene (*Os06g0229800*) encoding starch synthase II that played an important role in amylopectin biosynthesis was barley *SS*II*a* (*SS*II*-3*) gene. According to the isolation and characterization results of the two bi-parental *SS*II*a* alleles of the DH population, a 33-bp polymorphic difference in exon 2 was found (Figure 6). qRT-PCR analysis result indicated that the expression levels in each stage after flowering of *SS*II*a* allele in Naso Nijo were higher than those in TX9425 (Figure 7B). Furthermore, the expression pattern of *SS*II*a* alleles in our study was consistent to Radchuk et al. (2009). According to those results, we can conclude that the deletion was likely responsible for reducing *SS*II*a* transcript, thus resulting in a reduced amylopectin concentration in barley endosperm.

The conventional breeding method combining with MAS could significantly raise the breeding efficiency (Tian et al., 2010). In this study, a sequence-based marker (SSIIa-DNA) was designed according to the 33-bp difference between the *SS*II*a* alleles (Table S4). Amplified by primer SSIIa-DNA, two types (58 type-TX9425 and 124 type-Naso nijo) were separated within 182 barley varieties of the association mapping panel, in addition, there was a significant difference between the two types (Figure 7A). The result further indicated that the 33-bp nucleotide sequence was a functional polymorphic difference associated with grain amylopectin content and the developed molecular marker was suitable for MAS in barley breeding.

In summary, a QTL responsible for barley grain amylopectin content was detected in both bi-parental and association mapping panel respectively by linkage analysis and GWAS. A strong candidate for the *SS*II*a* gene and a functional polymorphism (a insertion/deletion of 33bp in exon 2) associated with different levels of grain amylopectin content were identified. Furthermore, a sequence-based molecular marker had been developed with potential application for MAS in breeding for desirable grain amylopectin content in barley.

## Author contributions

XF and JZ measured the grain amylopectin content, performed QTL, GWAS and candidate gene analysis, and wrote the manuscript. WD and YS prepared the grain flour for amylopectin content measurement and prepared the DNA and RNA of materials for relevant experiment. CL and BG managed the field experiments. RX supervised the project. All authors read and approved of the final manuscript.

### Conflict of interest statement

The authors declare that the research was conducted in the absence of any commercial or financial relationships that could be construed as a potential conflict of interest.

## Supplementary material

Table S1 Barley accessions for GWAS analysis.

Table S2 Descriptive statistics for amylopectin content of the DH population and two parents.

Table S3 Associated SNPs for grain amylopectin content for GWAS of single year separately.

Table S4 The primers for barley *SSIIa* gene sequencing, verification and expression analysis.

Table S5 The 64-bp Sequence of all SNPs identified and presented in Tables 3, 4.

Figure S1 Genetic linkage map of the DH population for QTL analysis.
