# Supplementary material for: Comparative Mapping and Candidate Gene Analysis of SSIIa Associated with Grain Amylopectin Content in Barley (Hordeum vulgare L.)
Source: Front Plant Sci. 2017 Sep 5;8:1531. doi: 10.3389/fpls.2017.01531 (PMC5591850; doi:10.3389/fpls.2017.01531)
Supplement: Table S1 — Barley accessions for GWAS analysis. [file Table1.DOCX]

Table S1 Barley accessions for GWAS analysis

| Accession Number | Barley  Germplasm  Name | Country  of  Origin | Row Type | Hulled/Naked | Spring/Winter |
| --- | --- | --- | --- | --- | --- |
| B001 | Sunong22 | China | Two-rowed | Hulled | Winter |
| B002 | Zhenong7 | China | Two-rowed | Hulled | Winter |
| B003 | Sunong91-712 | China | Two-rowed | Hulled | Winter |
| B004 | Yangnongpi4 | China | Two-rowed | Hulled | Winter |
| B005 | Chuangmai3 | China | Two-rowed | Hulled | Winter |
| B006 | Yangnongpi6 | China | Two-rowed | Hulled | Winter |
| B007 | Sunong16 | China | Two-rowed | Hulled | Winter |
| B008 | Huaai11 | China | Six-rowed | Naked | Winter |
| B009 | Yunpi1 | China | Two-rowed | Hulled | Winter |
| B010 | Yanyin1 | Japan | Two-rowed | Hulled | Winter |
| B011 | Aigan1 | China | Two-rowed | Hulled | Winter |
| B012 | 32122 | China | Two-rowed | Hulled | Winter |
| B013 | Zheda96-6 | China | Two-rowed | Hulled | Winter |
| B014 | Pudamai5 | China | Two-rowed | Hulled | Winter |
| B015 | Zhe08-49 | China | Six-rowed | Hulled | Winter |
| B016 | Edamai6 | China | Two-rowed | Hulled | Winter |
| B017 | Zhu05-043-2-1 | China | Two-rowed | Hulled | Winter |
| B018 | Sunong6472 | China | Two-rowed | Hulled | Winter |
| B019 | Suyinmai3 | China | Two-rowed | Hulled | Winter |
| B020 | Riyin1 | Japan | Two-rowed | Hulled | Winter |
| B021 | Riyin3 | Japan | Two-rowed | Hulled | Winter |
| B022 | Yangnongpi5 | China | Two-rowed | Hulled | Winter |
| B023 | Naso nijo | Japan | Two-rowed | Hulled | Winter |
| B024 | Yangpi1 | China | Two-rowed | Hulled | Winter |
| B025 | Pudamai9 | China | Two-rowed | Hulled | Winter |
| B026 | Yangnongpi8 | China | Two-rowed | Hulled | Winter |
| B027 | Harrinonijo | Japan | Two-rowed | Hulled | Winter |
| B028 | Zhexiu9 | China | Two-rowed | Hulled | Winter |
| B029 | Youzhi1 | China | Two-rowed | Hulled | Winter |
| B030 | Zhu9505-1-3 | China | Two-rowed | Hulled | Winter |
| B031 | Supi3 | China | Two-rowed | Hulled | Winter |
| B032 | 91dan2 | China | Two-rowed | Hulled | Winter |
| B033 | Shanxiliang2 | China | Two-rowed | Hulled | Winter |
| B034 | Tongmai10 | China | Two-rowed | Hulled | Winter |
| B035 | Yan99175 | China | Two-rowed | Hulled | Winter |
| B036 | Yangjian135 | China | Two-rowed | Hulled | Winter |
| B037 | Zhe35-21 | China | Two-rowed | Hulled | Winter |
| B038 | Edamai9706 | China | Two-rowed | Hulled | Winter |
| B039 | Zhu06049-1 | China | Two-rowed | Hulled | Winter |
| B040 | Hua11 | China | Two-rowed | Hulled | Winter |
| B041 | Hua22 | China | Two-rowed | Hulled | Winter |
| B042 | Yangsimai3 | China | Two-rowed | Hulled | Winter |
| B043 | Yangnongpi7 | China | Two-rowed | Hulled | Winter |
| B044 | Suhua2 | China | Two-rowed | Hulled | Winter |
| B045 | Haronanijo | Japan | Two-rowed | Hulled | Winter |
| B046 | Zhu96015-5-3 | China | Two-rowed | Hulled | Winter |
| B047 | Yuyaoxiangtian | China | Two-rowed | Hulled | Winter |
| B048 | Pudamai8 | China | Two-rowed | Hulled | Winter |
| B049 | 7204 | UK | Two-rowed | Hulled | Winter |
| B050 | Zhu97022 | China | Two-rowed | Hulled | Winter |
| B051 | Rudong6109 | China | Two-rowed | Hulled | Winter |
| B052 | Rudong5218 | China | Two-rowed | Hulled | Winter |
| B053 | Lian9723 | China | Two-rowed | Hulled | Winter |
| B054 | Hong07-456 | China | Two-rowed | Hulled | Winter |
| B055 | Hong08-718 | China | Two-rowed | Hulled | Winter |
| B056 | Zhepi33 | China | Two-rowed | Hulled | Winter |
| B057 | Edamai507 | China | Two-rowed | Naked | Winter |
| B058 | Zhu2005-7-9 | China | Two-rowed | Hulled | Winter |
| B059 | Yangsimai1 | China | Six-rowed | Hulled | Winter |
| B060 | Riyin4 | Japan | Six-rowed | Hulled | Winter |
| B061 | Supi4 | China | Two-rowed | Hulled | Winter |
| B062 | Xinan86-911 | China | Two-rowed | Hulled | Winter |
| B063 | Chuan52209 | China | Two-rowed | Hulled | Winter |
| B064 | Yangyin02 | China | Two-rowed | Hulled | Winter |
| B065 | Yanzhe3 | China | Two-rowed | Hulled | Winter |
| B066 | QS | China | Two-rowed | Hulled | Winter |
| B067 | Zhu3 | China | Two-rowed | Hulled | Winter |
| B068 | Zhu7 | China | Two-rowed | Hulled | Winter |
| B069 | Riyin2 | Japan | Six-rowed | Hulled | Winter |
| B070 | Zhouxuan1 | China | Two-rowed | Hulled | Winter |
| B071 | Hong09-784 | China | Two-rowed | Hulled | Winter |
| B072 | Zhu4 | China | Six-rowed | Hulled | Winter |
| B073 | Huadamai5 | China | Two-rowed | Hulled | Winter |
| B074 | Yunpi2 | China | Two-rowed | Hulled | Winter |
| B075 | Yunpi3 | China | Two-rowed | Hulled | Winter |
| B076 | Yunpi4 | China | Two-rowed | Hulled | Winter |
| B077 | Yunpi5 | China | Two-rowed | Hulled | Winter |
| B078 | Yunpi6 | China | Two-rowed | Hulled | Winter |
| B079 | Danyan | China | Two-rowed | Hulled | Winter |
| B080 | Hu01-2946 | China | Two-rowed | Hulled | Winter |
| B081 | Yunpi7 | China | Two-rowed | Hulled | Winter |
| B082 | Yunpi9 | China | Two-rowed | Hulled | Winter |
| B083 | Favorit | Hungary | Two-rowed | Hulled | Winter |
| B084 | Zhu5 | China | Two-rowed | Hulled | Winter |
| B085 | Hua2328 | China | Two-rowed | Hulled | Winter |
| B086 | Mei97-1455 | USA | Two-rowed | Hulled | Winter |
| B087 | Mei97-1338 | USA | Six-rowed | Naked | Winter |
| B088 | Zhu6 | China | Two-rowed | Naked | Winter |
| B089 | Huadamai6 | China | Two-rowed | Hulled | Winter |
| B090 | Huadamai7 | China | Two-rowed | Hulled | Winter |
| B091 | CM72 | USA | Six-rowed | Hulled | Winter |
| B092 | Gairdner | Australia | Two-rowed | Hulled | Winter |
| B093 | Frankin | Australia | Two-rowed | Hulled | Winter |
| B094 | T98189 | USA | Two-rowed | Hulled | Winter |
| B095 | Suyin27 | Japan | Two-rowed | Hulled | Winter |
| B096 | AcBacclm | USA | Six-rowed | Naked | Winter |
| B097 | AcBurman | USA | Six-rowed | Hulled | Winter |
| B098 | OR71 | USA | Six-rowed | Hulled | Winter |
| B099 | C2118 | USA | Six-rowed | Hulled | Winter |
| B100 | Dazhong8891 | China | Six-rowed | Hulled | Winter |
| B101 | Yangnongpi9 | China | Two-rowed | Hulled | Winter |
| B102 | Yangnongpi10 | China | Two-rowed | Hulled | Winter |
| B103 | Yangnongpi2 | China | Two-rowed | Hulled | Winter |
| B104 | Yangnongpi11 | China | Two-rowed | Hulled | Winter |
| B105 | Yangnongpi12 | China | Two-rowed | Hulled | Winter |
| B106 | Huangchangmang | China | Two-rowed | Naked | Winter |
| B107 | Hu1154 | China | Two-rowed | Hulled | Winter |
| B108 | Yang0187 | China | Two-rowed | Hulled | Winter |
| B109 | 1430R | China | Two-rowed | Hulled | Winter |
| B110 | Taixin9425 | China | Two-rowed | Hulled | Winter |
| B111 | Shijiazhuangdamai | China | Six-rowed | Hulled | Winter |
| B112 | Ningjindamai | China | Six-rowed | Hulled | Winter |
| B113 | Yixiandamai | China | Six-rowed | Hulled | Winter |
| B114 | 80-229 | China | Six-rowed | Hulled | Winter |
| B115 | Weixiancundamai | China | Six-rowed | Naked | Winter |
| B116 | Layiba | China | Six-rowed | Hulled | Winter |
| B117 | Jinancao | China | Six-rowed | Hulled | Winter |
| B118 | Ruodamai1 | China | Six-rowed | Naked | Winter |
| B119 | Manghuomai | China | Six-rowed | Hulled | Winter |
| B120 | Huodengmai | China | Six-rowed | Hulled | Winter |
| B121 | Maodamai1 | China | Six-rowed | Hulled | Winter |
| B122 | Changmangdamai | China | Six-rowed | Hulled | Winter |
| B123 | Mangdamai1 | China | Six-rowed | Hulled | Winter |
| B124 | Mangdamai2 | China | Six-rowed | Hulled | Winter |
| B125 | Silengmang | China | Six-rowed | Hulled | Winter |
| B126 | Damai1 | China | Six-rowed | Hulled | Winter |
| B127 | Tangtouda | China | Six-rowed | Hulled | Winter |
| B128 | Youzidamai | China | Six-rowed | Hulled | Winter |
| B129 | Damai2 | China | Six-rowed | Hulled | Winter |
| B130 | Sanyuehuang1 | China | Six-rowed | Naked | Winter |
| B131 | Ruodamai2 | China | Six-rowed | Naked | Winter |
| B132 | Duanmangdamai | China | Six-rowed | Hulled | Winter |
| B133 | Silengdamai | China | Six-rowed | Hulled | Winter |
| B134 | Changsuidamai | China | Six-rowed | Hulled | Winter |
| B135 | Liulengdamai1 | China | Six-rowed | Hulled | Winter |
| B136 | Tuheshang | China | Six-rowed | Naked | Winter |
| B137 | Midamai1 | China | Six-rowed | Naked | Winter |
| B138 | Duanmangdamai | China | Six-rowed | Naked | Winter |
| B139 | Laolaichuang | China | Six-rowed | Hulled | Winter |
| B140 | 58-40 | China | Six-rowed | Hulled | Winter |
| B141 | Zhisilengdamai | China | Six-rowed | Hulled | Winter |
| B142 | Jiangning1395 | China | Six-rowed | Hulled | Winter |
| B143 | Yangzhongbaimai | China | Six-rowed | Naked | Winter |
| B144 | Sanyuehuang2 | China | Six-rowed | Hulled | Winter |
| B145 | Liulengdamai2 | China | Six-rowed | Hulled | Winter |
| B146 | Yuexisanyuehuang | China | Six-rowed | Hulled | Winter |
| B147 | Qimengdamai | China | Six-rowed | Hulled | Winter |
| B148 | Jinxianluanqiangzi | China | Six-rowed | Hulled | Winter |
| B149 | Ximai | China | Six-rowed | Hulled | Winter |
| B150 | Bianzicaoda | China | Six-rowed | Hulled | Winter |
| B151 | Liudanhuai | China | Six-rowed | Naked | Winter |
| B152 | Chenghaidamai | China | Six-rowed | Hulled | Winter |
| B153 | Wudamai | China | Six-rowed | Hulled | Winter |
| B154 | Qinke | China | Six-rowed | Hulled | Winter |
| B155 | Pidamai | China | Six-rowed | Hulled | Winter |
| B156 | Longwupidamai | China | Six-rowed | Hulled | Winter |
| B157 | Damai3 | China | Six-rowed | Hulled | Winter |
| B158 | Hongmangdamai | China | Six-rowed | Hulled | Winter |
| B159 | Baidamai | China | Six-rowed | Hulled | Winter |
| B160 | Yangdamai1 | China | Six-rowed | Naked | Winter |
| B161 | Yangdamai2 | China | Six-rowed | Naked | Winter |
| B162 | Changmangruo | China | Six-rowed | Naked | Winter |
| B163 | Siyuehuang | China | Six-rowed | Hulled | Winter |
| B164 | Zhuchengmi | China | Six-rowed | Naked | Winter |
| B165 | Maodamai2 | China | Six-rowed | Hulled | Winter |
| B166 | Hongkedamai | China | Six-rowed | Hulled | Winter |
| B167 | Wumimai | China | Six-rowed | Naked | Winter |
| B168 | Damai4 | China | Six-rowed | Hulled | Winter |
| B169 | Zhouqudamai | China | Six-rowed | Hulled | Winter |
| B170 | Changyangmimai | China | Six-rowed | Naked | Winter |
| B171 | Xiaoguangcun | China | Six-rowed | Hulled | Winter |
| B172 | Changmaodamai | China | Six-rowed | Hulled | Winter |
| B173 | Guangtoudamai | China | Six-rowed | Hulled | Winter |
| B174 | Damai5 | China | Six-rowed | Hulled | Winter |
| B175 | Hezhanglaomai | China | Six-rowed | Naked | Winter |
| B176 | Midamai2 | China | Six-rowed | Naked | Winter |
| B177 | Toudenglaomai | China | Six-rowed | Hulled | Winter |
| B178 | Sansuimai | China | Six-rowed | Hulled | Winter |
| B179 | Maotoudamai | China | Six-rowed | Hulled | Winter |
| B180 | Hongsuimai | China | Six-rowed | Naked | Winter |
| B181 | Ruomai | China | Six-rowed | Naked | Winter |
| B182 | Liulengmai | China | Six-rowed | Hulled | Winter |
| B183 | Laomai | China | Six-rowed | Hulled | Winter |
| B184 | Zang3213 | China | Six-rowed | Hulled | Winter |
| B185 | Zhongpin1066 | China | Six-rowed | Hulled | Winter |
