# Supplementary material for: Comparative Mapping and Candidate Gene Analysis of SSIIa Associated with Grain Amylopectin Content in Barley (Hordeum vulgare L.)
Source: Front Plant Sci. 2017 Sep 5;8:1531. doi: 10.3389/fpls.2017.01531 (PMC5591850; doi:10.3389/fpls.2017.01531)
Supplement: Table S2 — Descriptive statistics for amylopectin content of the DH population and two parents. [file Table2.DOCX]

Table S2 Descriptive statistics for amylopectin content of the DH population and two parents.

| Traits | Environment | Parents | | | DH lines | | | |
| --- | --- | --- | --- | --- | --- | --- | --- | --- |
|  |  | Naso Nijo | TX9425 | T-value | Mean | Range | SD | CV |
| Amylopectin  Content | YZ(13-14) | 48.25 | 41.79 | 58.32** | 45.01 | 39.77-51.51 | 2.03 | 4.50 |
|  | YC(14-15) | 47.89 | 42.05 | 44.06** | 45.34 | 35.32-55.42 | 2.56 | 5.65 |

*, **: significantly different between parents at 0.05 and 0.01 levels, respectively
