# Supplementary material for: Comparative Mapping and Candidate Gene Analysis of SSIIa Associated with Grain Amylopectin Content in Barley (Hordeum vulgare L.)
Source: Front Plant Sci. 2017 Sep 5;8:1531. doi: 10.3389/fpls.2017.01531 (PMC5591850; doi:10.3389/fpls.2017.01531)
Supplement: Table S3 — Associated SNPs for grain amylopectin content for GWAS of single year separately. [file Table3.DOCX]

Table S3 Associated SNPs for grain amylopectin content for GWAS of single year separately

| Traits | Marker | Chr. | Position(cM) | P-value | R^2^(%) | Annotation |
| --- | --- | --- | --- | --- | --- | --- |
| Amylopectin Content  (2014-2015) | SNP0988 | 1 | 25.91 | 9.08E-04 | 10.09 | NA |
|  | SNP1472 | 1 | 47.82 | 4.17E-04 | 9.33 | NA |
|  | SNP2080 | 2 | 115.36 | 5.85E-04 | 8.65 | MLOC_22418.1, SAUR-like auxin-responsive protein family |
|  | SNP0187 | 3 | 90.33 | 3.09E-04 | 8.03 | AK252941.1, Protein kinase |
|  | SNP3120 | 7 | 70.68 | 3.69E-04 | 10.02 | MLOC_62313.1, Endo-1,4-beta-glucanase |
| Amylopectin Content  (2015-2016) | SNP0336 | 1 | 49.58 | 2.85E-04 | 8.28 | NA |
|  | SNP0600 | 3 | 68.20 | 8.90E-04 | 9.36 | MLOC_26433.1, maternal effect embryo arrest 22 |
|  | SNP3120 | 7 | 70.68 | 3.23E-04 | 9.06 | MLOC_62313.1, Endo-1,4-beta-glucanase |
|  | SNP3630 | 7 | 140.86 | 1.16E-04 | 11.21 | NA |
