# Supplementary material for: Comparative Mapping and Candidate Gene Analysis of SSIIa Associated with Grain Amylopectin Content in Barley (Hordeum vulgare L.)
Source: Front Plant Sci. 2017 Sep 5;8:1531. doi: 10.3389/fpls.2017.01531 (PMC5591850; doi:10.3389/fpls.2017.01531)
Supplement: Table S4 — The primers for barley SSIIa gene sequencing, verification and expression analysis. [file Table4.DOCX]

Table S4 The primers for barley *SSⅡa* gene sequencing, verification and expression analysis.

| Prime | Prime sequence |
| --- | --- |
| SSⅡa-1 | F: CTGCCGTTTGTTGTGCC  R: CCCGCAACATCTCCAAG |
| SSⅡa-2 | F: GTCGTCGTTGCTGCTGA  R: CTGTCCAGCAGCCTTGT |
| SSⅡa-3 | F: CCTACGATGTCGGAGTCC  R: ACTGGTTGGCGTTGGAG |
| SSⅡa-4 | F: TTCGCCTTCCGTTCAGT  R: CGTTCCCATCAAGAAGGAC |
| SSⅡa-5 | F: CCTATGTGCGTGCTTGA  R: CAGGGAGAAGTTGGTGTAG |
| SSⅡa-DNA | F: CATCAGTAACAAGGTGCCG  R: GCAATGCCCATACCTGTTT |
| SSⅡa-RNA | F: CCATCAGTAACAAGGTGCCG  R: GCCGAAAGAGCCTTTGGTT |
| SSⅡa-RT | F: GCCCATCTGTCAATCCA  R: GCGGCACCTTGTTACTG |
