# Supplementary material for: Comparative Mapping and Candidate Gene Analysis of SSIIa Associated with Grain Amylopectin Content in Barley (Hordeum vulgare L.)
Source: Front Plant Sci. 2017 Sep 5;8:1531. doi: 10.3389/fpls.2017.01531 (PMC5591850; doi:10.3389/fpls.2017.01531)
Supplement: Table S5 — The 64-bp Sequence of all SNPs identified and presented in Tables 3, 4. [file Table5.DOCX]

Table S5 The 64-bp Sequence of all SNPs identified and presented in table 3 and 4

| SNP Marker | 64-bp Sequences |
| --- | --- |
| SNP1920 | TGCAGGGTACAAGAGGGGAGCCCTGAGGGCATGCTGCGGAGGCGGTGGCCCATACAACTACAAC |
| SNP2508 | TGCAGTTCTTGCTTTAGCATGCGAAAATTAAGGTCCTGGTGTATGCATGTATCTTGCACTTGCA |
| SNP0788 | TGCAGCATGACGAGTTTGTCATTTACACTGACAATAAAAGCTTGATTCATCTCGAGGAACAACA |
| SNP2137 | TGCAGTAAAAACGGATCGTACGTACATATATACGTACATTAGATTTGGTCTACCGTGGAGTAAT |
| SNP1472 | TGCAGCAGCCCCCCGAACCCGCCGAGCAGCAGGTCGCCGTAGGCGCTGAGGTCTGTTAGGAATG |
| SNP0336 | TGCAGACAGTGTGAGACAAACGGCTAAATAGGCAAGTACTACTAACATCTATACACAGTCATCA |
| SNP0057 | TGCAGAAAGGGCCCCTCCTATGTGCCGCTTACTACCGCAAAGAGGCGCAGCTTCACACAGGAAA |
| SNP2489 | TGCAGCGGTCGCCTTCGTCTCCGCCAAGGTTCCAGGCGGTGTTCGCCTGCTGCTACTGCCCCAG |
| SNP0600 | TGCAGAGCCATGCAAAGGCAAGTTTGCATTCGGCACCTAATAAAAGCAGGCTAAATCAAATGTC |
| SNP2706 | TGCAGCTCATTCATCAAGGCTACAGCAGCTGCGACGTCAAACCCATAATCATGATCATCGCCGT |
| SNP4404 | TGCAGGTGCTCCTCCGCGACTCTTGCGATGAAGAGGCCGAGCGTCGGGAAGGAGCGGAAGCCAA |
| SNP4298 | TGCAGGTCGAGGCGGAGCAGGTCGCGCACCCGCACGTAGCCATCGCTCCTCATGTCCAGCCTCA |
| SNP3654 | TGCAGGCCGTGGCGCTCCGCCGCCAGCCAGCTCCCACCATCGCGGCTCCCCTGGATCGCGTTCG |
| SNP3120 | TGCAGCTTGCGCCGCAGGAACCCCCTGGAGAGCAGCACCTTGGTGCCGACCCTCTTGTCGTCCC |
| SNP3630 | TGCAGGCCGACCTCTCCCGCTTCGGTCACGACTGGCGTCAGGCGACCTCCATCTTCTTCTCTAA |
