# Supplementary figures and images for: Comparative Mapping and Candidate Gene Analysis of SSIIa Associated with Grain Amylopectin Content in Barley (Hordeum vulgare L.)
Source: Front Plant Sci. 2017 Sep 5;8:1531. doi: 10.3389/fpls.2017.01531 (PMC5591850; doi:10.3389/fpls.2017.01531)

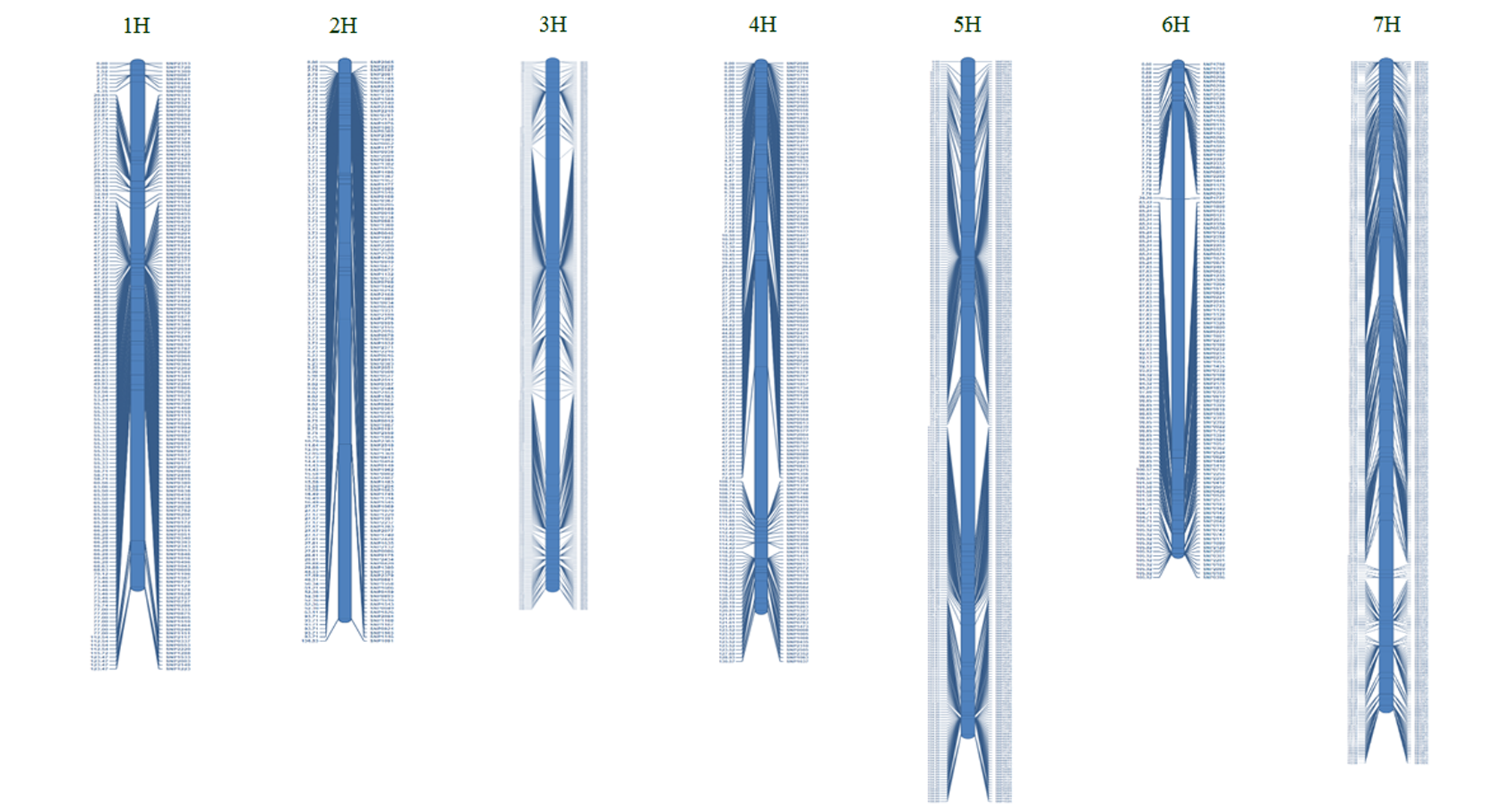

Supplement: Figure S1 — Genetic linkage map of the DH population for QTL analysis. [file Image1.TIF]
